# Cerebral Metastasis of a Malignant Pleural Mesothelioma: A Case Report and Review of the Literature

**DOI:** 10.7759/cureus.241

**Published:** 2015-01-15

**Authors:** Aswin Chari, Angelos G Kolias, Kieren Allinson, Thomas Santarius

**Affiliations:** 1 Neurosurgery, Imperial College London & Imperial College NHS Trust; 2 Clinical Neurosciences, Addenbrooke's Hospital & University of Cambridge; 3 Pathology, University of Cambridge; 4 Neurosurgery, Cambridge University Hospitals

**Keywords:** mesothelioma, brain metastasis, spine metastasis, metastasis, neurosurgery, neuro-oncology, radiation oncology

## Abstract

Background: Malignant pleural mesothelioma (MPM) is an aggressive malignant neoplasm that was thought to be a localised disease with limited metastatic capability. However, recent post-mortem studies have identified metastases to the central nervous system (CNS) in about 3% of cases.

Case Description: We present the case of a 65-year-old with a solitary supratentorial metastatic deposit of MPM treated with surgical resection and adjuvant whole brain radiotherapy. Despite a good surgical outcome with symptomatic recovery, the patient died of cardiopulmonary compromise five months postoperatively.

Conclusions: Although rare, CNS metastasis of MPM is a condition that neurosurgeons should be aware of. CNS metastases may occur via three distinct mechanisms, namely perineural spread, leptomeningeal carcinomatosis and, most commonly, haematogenous spread leading to parenchymal deposits. Surgical resection of these deposits can lead to symptomatic improvement, and together with radiotherapy, to local disease control. However, the overall survival remains poor.

## Introduction

Malignant mesothelioma is an aggressive malignant neoplasm that can arise from mesothelial cells of the pleura, peritoneum, pericardium, and tunica vaginalis of the testis. The main cause of malignant pleural mesothelioma (MPM) is asbestos exposure, which can occur as an occupational hazard, for example, in ship builders and construction workers. Presentation usually trails exposure by 20-30 years [1].

For many years, MPM was thought to be a localised disease with limited metastatic capability, but a large post-mortem study of 318 MPM patients has identified that spread to the contralateral thoracic cavity and distant sites can be seen in a majority [2]. Metastases to the central nervous system (CNS), however, are rare, occurring in about 3% of post-mortem cases [2-3].

We present the case of a solitary supratentorial metastatic deposit and discuss the treatment strategy. We also conducted a literature review by searching the MEDLINE database for “malignant mesothelioma AND (central nervous system OR brain OR spinal cord)". We examined the three purported mechanisms of spread of MPM to the CNS and potential treatment options for each, specifically looking at the treatment strategies and overall survival of patients with hemispheric metastases.

Informed consent from the patient’s next of kin was sought and obtained.

## Case presentation

**Figure 1:**
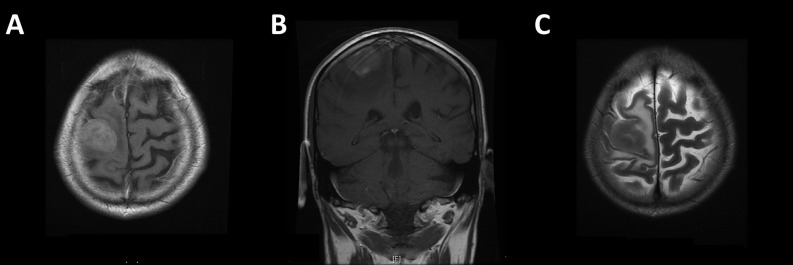
MR images showing solitary intrinsic lesion centred around the pre-central gyrus, confirmed to be metastatic deposit of MPM on histological assessment. A/B: Post-gadolinium T1-weighted images; C: T2-weighted image

**Figure 2:**
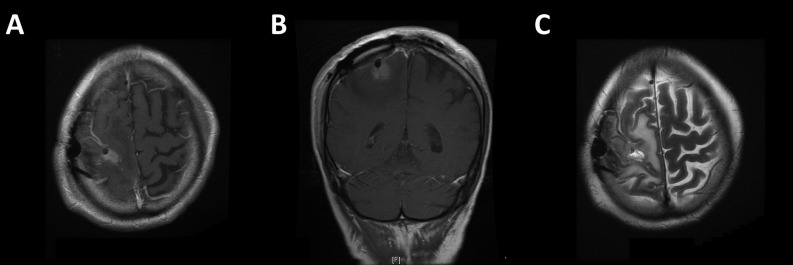
Postoperative MR images confirming gross total resection. A/B: Post-gadolinium T1-weighted images showing some hyperintense material in the cavity that does not enhance compared to pre-contrast sequence; C: T2-weighted image

**Figure 3:**
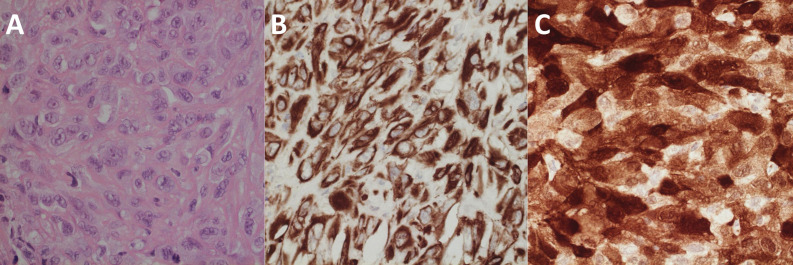
Histological images confirming diagnosis of MPM. A: light microscopy with haematoxylin & eosin stain showing a malignant neoplasm featuring sheets of cohesive epithelioid and plump spindle cells with large nuclei, lobulated nuclear outlines and prominent nucleoli. There were multiple foci of tumour necrosis and conspicuous mitotic activity. No glandular or squamous differentiation was identified. B/C: Immunohistochemical preparations showing diffuse and strong positivity for broad-spectrum cytokeratin AE1/3 (B) and patchy but strong staining for calretinin (C). There was also patchy but strong staining for cytokeratin 5/6 and epithelial membrane antigen with only weak focal staining for D2-40.

A 65-year-old left-handed gentleman presented to the emergency department with a focal seizure involving the left upper limb. His only past medical history of note was a left malignant pleural mesothelioma for which he had completed six cycles of pemetrexed and cisplatin chemotherapy one year previously. It had shown excellent partial radiological response and was now under surveillance with stable appearances on follow-up computed tomography (CT) scans. Neurological examination revealed isolated left upper limb weakness (4/5 on MRC scale) and an unsteady gait. CT and magnetic resonance (MR) imaging revealed a heterogeneously enhancing intrinsic lesion centred around the right pre-central gyrus measuring 22 mm x 30 mm x 20 mm with extensive surrounding oedema (Figure 1), highly suspicious of a solitary metastasis. Staging CT revealed stable lung disease in the thorax and no evidence of any new primary cancer. The patient was commenced on high dose dexamethasone and phenytoin, and surgical resection was planned. Preoperative neurological examination showed progression of his left hemiparesis to hemiplegia. He underwent gross total resection, confirmed on postoperative MR imaging (Figure 2). Light microscopy and immunohistochemistry of the excised lesion confirmed the suspected diagnosis of metastatic mesothelioma (Figure 3).

After an uncomplicated recovery, he was discharged on postoperative day three. At follow-up on postoperative day 10, he was able to mobilise without a frame and went on to receive 20 Gy of whole brain radiotherapy in five fractions. Despite this, he died of cardiopulmonary compromise from his mesothelioma five months later.

Informed consent from the patient’s next of kin (wife) was sought and obtained for the publication of this case report. 

## Discussion

We describe the case of a solitary metastatic deposit of MPM with overall survival of five months following successful surgical resection and adjuvant whole brain radiotherapy. Given the increasing incidence of the disease and the recognition that up to 3% of patients may have CNS metastasis, it is important to consider the different mechanisms of metastasis of MPM to the CNS and potential treatment strategies for each.

Three distinct mechanisms for metastatic spread to the CNS have been described in the literature. The first is via perineural spread resulting in nerve root and spinal cord compression [4-9]. Whilst one study reported successful gross total resection of an intradural extramedullary lesion [8], other authors reported intramedullary tumour with subtotal resection [4, 6-7, 10]. In general, authors report some functional recovery and recommend surgical decompression for such cases, although in one case, stereotactic radiosurgery was used to palliate symptoms [10]. Median overall survival after diagnosis of spinal cord compression is reported at 75-360 days [11].

The second mechanism is haematogenous spread leading to diffuse leptomeningeal carcinomatosis [12-13]. The diagnosis is made by contrast-enhanced magnetic resonance imaging and cytology from cerebrospinal fluid. Neither of the two cases reported in the literature discuss successful treatment strategies [12-13].

The third, and most common, mechanism is haematogenous spread leading to solitary metastases to the supra- and infra-tentorial compartments [11]. Solitary CNS metastases were first reported in 1983 [14]. In 1991, a large post-mortem study found CNS metastases in 3% of 171 mesotheliomas [3], findings that have been recently corroborated [2].

The first report of successful neurosurgical intervention for solitary CNS metastasis of MPM was in 1995, where gross total resection of a solitary right frontal lesion followed by whole brain and local radiotherapy was found to improve neurological symptoms and result in a 36-month recurrence-free survival [15]. Since then, a number of studies have reported intervention for cerebral metastases of MPM (Table 1) [15-19]. Treatment strategies have been highly heterogeneous, and overall survival has been poor despite aggressive management (Table 1). However, the published cases, including the current case, suggest that the cause of death usually surrounds progression of thoracic disease with low surgical morbidity and effective symptom control from neurosurgical intervention. This supports the case for treating cerebral metastases. A recent review puts median overall survival for solid brain metastases at 83-183 days [11], which concurs with the cases identified in the literature, as seen in Table 1.

**Table 1:** Case reports of active management of cerebral metastases of malignant pleural mesothelioma. Case reports of active management of cerebral metastases of malignant pleural mesothelioma [15-23] * Indicates alive at the time of reporting.

Paper	Age	Sex	Number of Deposits	Treatment Strategy	Survival	Notes
Kitai, et al. 1995	62	M	Single	Gross total resection + whole brain radiotherapy	36 months*	
Wronski, et al. 1993	52	F	Single	Gross total resection	10 days	Cause of death: Constrictive pericardial disease
Hurmuz, et al. 2009	56	F	Multiple	Whole brain radiotherapy	1 month	Cause of death: Respiratory failure
Colleoni, et al. 1996	55	M	Multiple	Chemotherapy with lomustine, carboplatin, vinorelbine, fluorouracil and folates	Unknown	
Krishnaraj, et al. 2003	64	M	Single	Gross total resection + radiotherapy	Unknown	Palliative radiotherapy
Mah, et al. 2004	67	M	Two	Gross total resection	3 months*	
Ishikawa, et al. 2010	56	M	Single	Gross total resection + whole brain radiotherapy	7 months*	Recurrence after 7 months, repeat surgery performed
Winfree, et al. 2004	71	F	Single	Gross total resection + whole brain radiotherapy + chemotherapy with adriamycin and cisplatin	8 months	Cause of death: Cardiopulmonary arrest
Hortobagyi, et al. 2008	71	M	Multiple	Debulking largest mass	Unknown	
This case	65	M	Single	Gross total resection + whole brain radiotherapy	5 months	Cause of death: Respiratory failure

## Conclusions

CNS metastasis of MPM is rare but a condition that neurosurgeons should be aware of. Surgical resection of symptomatic solitary metastases can lead to symptomatic improvement, and together with radiotherapy, to local disease control. However, the overall survival remains poor. 
